# Network analysis of psychological and behavioral variables in firefighters: identification of central nodes and bridge variables

**DOI:** 10.3389/fpubh.2026.1846200

**Published:** 2026-06-03

**Authors:** Cuiqing Zhao, Huiyu Wang, Guoqing Zhu, Tongling Wang, Wenjia Chen

**Affiliations:** 1 School of Sports Science, Nantong University, Nantong, China; 2 School of Safety Engineering, China University of Mining and Technology, Xuzhou, China; 3 School of Physical Education, China University of Mining and Technology, Xuzhou, China

**Keywords:** bridge centrality, firefighters, job stress, network analysis, occupational burnout, social support

## Abstract

**Objective:**

This study employed network analysis to systematically explore the complex associative structure among 14 psychological and behavioral variables in firefighters, including burnout, job stress, work–family conflict, social support, sleepiness, self-efficacy, and physical activity. The aim was to identify central nodes and cross-system bridge variables to inform targeted psychological interventions.

**Methods:**

A sample of 637 Chinese firefighters was recruited. Fourteen psychological and behavioral variables were included to construct a psychological network model using the EBICglasso regularized partial correlation method. Strength centrality, closeness centrality, betweenness centrality, and expected influence were calculated to identify central nodes. Bridge centrality indices were computed to identify cross-system bridge variables. Network stability was assessed using non-parametric bootstrap and case-dropping bootstrap procedures.

**Results:**

The strongest edge weight in the network was between emotional exhaustion and depersonalization (weight = 0.676), followed by work interference with family and family interference with work (weight = 0.633), interpersonal stress and role stress (weight = 0.535), and significant-other support and friend support (weight = 0.512). The node with the highest strength centrality was emotional exhaustion (1.29), followed by depersonalization (0.85) and interpersonal stress (0.70). Bridge centrality analysis revealed that reduced personal accomplishment (bridge strength = 0.581), family interference with work (0.558), and self-efficacy (0.525) exhibited high bridge strength, while sleepiness (bridge expected influence = 0.477) served as the key bridge variable connecting other psychological systems. Robustness checks indicated that the correlation stability (CS) coefficients for strength centrality and expected influence were both 0.75 (exceeding the 0.50 benchmark), confirming the stability and reliability of the network structure.

**Conclusion:**

Emotional exhaustion represents the core feature in the psychological problem network of firefighters. Sleepiness and self-efficacy serve as key bridge variables connecting different psychological systems. These findings suggest that emotional exhaustion, self-efficacy, and sleepiness may serve as empirically prioritized candidates for future longitudinal studies and targeted intervention research aimed at promoting firefighter mental health.

## 1 Introduction

Firefighters, as a core force in emergency rescue, are chronically exposed to high-intensity, high-risk occupational environments. Tasks such as fire suppression, natural disaster rescue, and major accident response frequently expose firefighters to traumatic events, subjecting them to psychological stress far exceeding that of the general working population (1). Research has demonstrated that psychological problems such as occupational burnout and post-traumatic stress disorder (PTSD) symptoms are particularly prevalent among firefighters (2), and these mental health issues not only impair firefighters’ own physical and psychological well-being (3) but may also compromise the quality of their daily training and emergency response capabilities in real-world operations, thereby threatening public safety. Occupational burnout is one of the most widely studied mental health concerns in the firefighter population. Maslach and Leiter’s (4) three-dimensional burnout model conceptualizes burnout as comprising emotional exhaustion, depersonalization, and reduced personal accomplishment. Emotional exhaustion manifests as feeling depleted of energy due to excessive consumption of psychological resources; depersonalization reflects cynical and detached attitudes toward work recipients; and reduced personal accomplishment entails negative self-evaluation of one’s work value and competence. Among rescue workers, the prevalence of occupational burnout has been reported to be as high as 57% (5), and it is closely associated with job stress, work–family conflict, and sleep disturbances (6, 7).

Job stress is a significant antecedent variable affecting firefighter mental health. A systematic review indicated that sources of job stress among firefighters are diverse, encompassing factors such as family responsibilities, sleep disorders, and maladaptive coping strategies (8). High levels of job stress not only directly exacerbate burnout symptoms but may also indirectly impair firefighters’ mental health through pathways such as triggering work–family conflict (9) and undermining perceived social support (10). Work–family conflict, defined as the tension between work and family roles, is particularly salient among firefighters, as shift schedules, 24-h on-call requirements, and unpredictable emergency deployments make it difficult for them to fulfill family responsibilities (3). Social support is widely recognized as an important protective factor that buffers occupational stress and prevents psychological problems. Perceived social support encompasses three dimensions—support from family, friends, and significant others—and provides individuals with emotional comfort, informational resources, and tangible assistance, thereby enhancing their capacity to cope with stress (11). For firefighters, support from colleagues and supervisors is especially important, as they are most likely to understand the unique challenges of firefighting work (12). Sleepiness and sleep problems represent another noteworthy health risk factor in the firefighter population; sleepiness is associated not only with cognitive decline and difficulties in emotion regulation but may also increase the risk of occupational injuries (13). Furthermore, the relationship between sleep problems and occupational burnout is bidirectional: burnout may lead to deteriorated sleep quality, while sleep insufficiency may exacerbate emotional exhaustion and reduced work efficacy (6, 14). Self-efficacy refers to an individual’s subjective belief in their capacity to organize and execute the courses of action required to accomplish a specific behavior (15). It reflects confidence in one’s ability to regulate motivation, behavior, and the social environment, and this cognitive self-appraisal influences the goals individuals pursue, the effort they invest in achieving those goals, and their actual performance at specific behavioral levels. Given that firefighters frequently confront hazardous situations that may challenge the limits of their capabilities, their level of self-efficacy constitutes a particularly valuable cognitive resource (16). Research has shown that among rescue workers (including firefighters and paramedics), perceived stress is significantly associated with quality of life only among individuals with low self-efficacy, whereas this association is absent among those with high self-efficacy, suggesting that self-efficacy plays an important role in the relationship between job stress and mental health (17).

Traditional psychological research has predominantly employed regression analysis, structural equation modeling, or mediation–moderation models to examine relationships among variables. Although these methods effectively address specific hypothesis-driven questions, they typically presuppose causal directions and hierarchical structures among variables, making it difficult to comprehensively capture the complex interactions among multiple variables. In recent years, network analysis has been introduced to the field of psychological research as an emerging data-driven approach, offering a novel perspective for understanding the complex relationships among psychological and behavioral variables (18, 19). In psychological network models, each psychological variable is represented as a node, and the conditional dependence relationships between variables are represented as edges, with edge thickness reflecting the strength of the partial correlation between two variables after controlling for all other variables (20). By computing centrality indices, researchers can identify the most influential central nodes in the network—nodes that exhibit the strongest conditional associations with other nodes throughout the network (21). It is important to distinguish between two types of influential nodes in network analysis: central nodes and bridge nodes. Central nodes are those with high overall connectivity within the network, meaning they exhibit the strongest conditional associations with other variables across the entire network (21). Bridge nodes, by contrast, may have lower within-community connectivity but serve as critical cross-system connectors. They link distinct symptom clusters and are hypothesized to play key roles in the co-occurrence and linkage of psychological problems from one domain to another (22). For example, Zhang et al. demonstrated this distinction in a network analysis of anxiety and depression symptoms in Chinese disabled older adults, showing that variables serving as bridges between the two symptom systems were conceptually and empirically distinct from those with the highest within-network centrality (23). This distinction holds particular significance for firefighter mental health research, as the psychological problems faced by firefighters typically involve multiple systems (e.g., burnout, stress, and social support systems), and understanding which variables serve as critical bridges rather than simply which are most central is essential for designing comprehensive intervention programs.

The application of network analysis in occupational mental health research has been steadily increasing. Zhang et al. (24) employed network analysis to explore the relationships among mental health, recovery experiences, sleep, and fatigue in firefighters, finding that mental health problems constituted the central node in the network and that sleep quality was more strongly associated with mental health problems than sleep duration. Cheng et al. (25) conducted a network analysis of PTSD and depressive symptoms in firefighters, revealing unique connectivity patterns between specific symptoms. Liu et al. (14) recently performed a network analysis of burnout and sleep disturbances in firefighters and found that subjective sleep quality was the central node in the network, while emotional exhaustion and daytime dysfunction served as key bridge nodes connecting the two symptom clusters. However, existing studies have predominantly focused on the network relationships among two or three psychological constructs, and few have simultaneously incorporated the wide range of psychological and behavioral variables confronting firefighters—including three dimensions of occupational burnout, three dimensions of job stress, bidirectional work–family conflict, three sources of social support, sleepiness, self-efficacy, and physical activity—into a single comprehensive network model. This lack of a comprehensive perspective has limited our understanding of the full picture of firefighters’ psychological and behavioral problems and constrained the design of targeted intervention programs.

Based on the above research background and gaps, the present study recruited Chinese firefighters as participants and simultaneously incorporated 14 psychological and behavioral variables spanning occupational burnout, job stress, work–family conflict, social support, sleepiness, self-efficacy, and physical activity to construct an EBICglasso regularized partial correlation network model. The study aimed to: (1) reveal the overall network structure and key connections among multiple psychological and behavioral variables in firefighters; (2) identify the most influential central nodes in the network through centrality indices; (3) identify key bridge variables involved in cross-system transmission through bridge centrality indices; and (4) provide evidence-based theoretical guidance for targeted interventions to promote firefighter mental health.

## 2 Methods

### 2.1 Participants

This study employed a stratified cluster random sampling method and conducted the survey via an online questionnaire platform between November and December 2025. Inclusion criteria were: (1) active-duty fire and rescue personnel; (2) aged ≥ 18 years; and (3) provision of informed consent and voluntary participation. Exclusion criteria were: (1) continuous leave of absence exceeding 3 months due to injury or illness; and (2) incomplete responses or evidence of patterned responding. After excluding invalid questionnaires characterized by excessively short completion times, fixed response patterns, or missing key information, the final valid sample was obtained. This study was approved by the Ethics Committee of Xuzhou First People’s Hospital (approval number: 2025-KY-064), and all participants provided written informed consent. The demographic characteristics of the sample are as follows: participants ranged in age from 18 to 59 years, with a mean age of 26.90 years (*SD* = 7.20), predominantly comprising young adults. Regarding educational attainment, 126 participants (19.8%) had a high school education or below, 165 (25.9%) had a technical secondary school or vocational high school diploma, 298 (46.8%) had a junior college or bachelor’s degree, and 48 (7.5%) held a master’s degree or above, with over 90% having a junior college or bachelor’s degree or below. Concerning household registration type, 533 participants (83.7%) held urban household registration and 104 (16.3%) held rural household registration, with the vast majority being urban registrants. This age profile is generally consistent with the overall demographic composition of China’s professional fire and rescue workforce, which is predominantly composed of young adults due to the extreme physical demands of the profession. However, the sample demonstrated a high concentration of urban registrants.

### 2.2 Measures

#### 2.2.1 Occupational burnout

The Maslach Burnout Inventory–Human Services Survey (MBI-HSS) was used to measure firefighters’ occupational burnout levels (4). This scale consists of 22 items rated on a 7-point Likert scale (0 = never, 6 = every day) and comprises three dimensions: emotional exhaustion (9 items; e.g., “I feel emotionally drained from my work,” “I feel fatigued when I get up in the morning and have to face another day on the job”), depersonalization (5 items; e.g., “I’ve become more callous toward people since I took this job”), and reduced personal accomplishment (8 items, all reverse-scored; e.g., “I feel I’m positively influencing other people’s lives through my work”). Higher scores on each dimension indicate more severe burnout symptoms; the personal accomplishment dimension is reverse-scored, with higher scores indicating lower accomplishment. In the current sample, Cronbach’s *α* for this scale was 0.88.

#### 2.2.2 Job stress

An adapted 12-item occupational stress scale, tailored specifically for the high-intensity context of emergency rescue personnel and rated on a 5-point Likert scale (1 = strongly disagree, 5 = strongly agree), was used to measure perceived job stress levels. Rather than using generic life stress measures, this instrument draws upon established occupational stress frameworks, specifically role stress theory (26) and the Job Demands-Resources model (27), to capture context-specific stressors. The scale includes three dimensions: task and control stress (e.g., “My workload exceeds what I can handle,” “I feel unable to control the pace and progress of my work”), role and development stress (e.g., “The role demands at work make me feel conflicted,” “Issues of promotion and compensation at work make me feel anxious”), and interpersonal and emotional depletion stress (e.g., “Interpersonal relationships at work cause me distress,” “Prolonged high-intensity work leaves me physically and mentally exhausted and emotionally depressed”). Higher scores on each dimension indicate greater job stress of that type. In the current sample, this scale demonstrated excellent internal consistency (Cronbach’s *α* = 0.98).

#### 2.2.3 Work–family conflict

The Work–Family Conflict Scale (18 items) was used to measure two dimensions: work interference with family (WIF) and family interference with work (FIW) (28), each comprising 9 items rated on a 5-point Likert scale (1 = strongly disagree, 5 = strongly agree). Representative WIF items include “The time demands of my work make it difficult to attend to family activities” and “Work stress makes me feel emotionally low and impatient at home.” Representative FIW items include “Family matters distract me at work” and “Fatigue from family responsibilities affects my work efficiency.” Higher scores on each dimension indicate greater interference in the respective direction. In the current sample, Cronbach’s *α* for this scale was 0.98.

#### 2.2.4 Perceived social support

The Multidimensional Scale of Perceived Social Support (MSPSS) was used to measure firefighters’ perceived social support levels (29), comprising 12 items rated on a 7-point Likert scale (1 = very strongly disagree, 7 = very strongly agree). The scale includes three dimensions with 4 items each: significant-other support (e.g., “There is a special person who is around when I am in need”), family support (e.g., “I can get emotional support from my family when I need it,” “My family understands my feelings”), and friend support (e.g., “My friends can share my joys and sorrows”). Higher scores on each dimension indicate more adequate perceived social support from that source. In the current sample, Cronbach’s *α* for this scale was 0.97.

#### 2.2.5 Sleepiness

The Epworth Sleepiness Scale (ESS) was used to assess the severity of daytime excessive sleepiness in firefighters (30). The scale comprises 8 everyday situations (e.g., “sitting and reading,” “sitting quietly in a public place,” “in a car while stopped in traffic for a few minutes”), and respondents rate the likelihood of dozing off in each situation on a 4-point scale (0 = would never doze, 3 = high chance of dozing). Total scores range from 0 to 24, with scores ≥ 10 indicating excessive sleepiness and scores ≥ 15 indicating severe sleepiness. Higher scores indicate more pronounced daytime sleepiness symptoms. In the current sample, Cronbach’s *α* for this scale was 0.89.

#### 2.2.6 Self-efficacy

An adapted Chinese version of the General Self-Efficacy Scale (GSES) was used to assess self-efficacy in firefighters (31), with item wording adjusted to suit firefighter training contexts. The scale comprises 9 items (e.g., “Even when encountering setbacks, I can quickly adjust and continue to work hard,” “I can effectively cope with various pressures during training”), rated on a 10-point Likert scale (1 = no confidence, 10 = absolute confidence). Higher scores indicate stronger self-efficacy. In the current sample, this scale demonstrated excellent internal consistency (Cronbach’s *α* = 0.96).

#### 2.2.7 Physical activity

An adapted Chinese version of the Physical Activity Rating Scale-3 (PARS-3) was used to assess physical activity levels (32). The scale covers exercise intensity (1–3 points; e.g., 1 = light activities such as walking or tai chi, 3 = vigorous activities such as fast running or basketball), exercise frequency (1–4 points; from 1–3 times per month to ≥ 5 times per week), and exercise duration per session (1–4 points; from < 30 min to ≥ 90 min). The total energy expenditure score is calculated as the product of the three dimensions (maximum 48 points), with higher scores indicating higher physical activity levels. Because the PARS-3 total score is computed as a product, it naturally produces a right-skewed distribution. In the current sample, the physical activity score showed positive skewness (1.586) and excess kurtosis (2.236).

### 2.3 Statistical analysis

The EBICglasso method was employed to estimate the regularized partial correlation network (20). This method applies the graphical LASSO for regularized estimation of the partial correlation matrix and uses the Extended Bayesian Information Criterion (EBIC; hyperparameter *γ* = 0.5) to select the optimal tuning parameter *λ*, shrinking potentially spurious weak edges to zero to yield a sparse and interpretable network structure. To account for non-normality in ordinal or skewed variables (e.g., physical activity), network estimation utilized the cor_auto function from the qgraph package. Network visualization utilized the Fruchterman–Reingold algorithm layout, with green and red edges representing positive and negative partial correlations, respectively, and edge thickness reflecting the magnitude of partial correlations. The 14 observed variables were assigned to four communities: the burnout system (emotional exhaustion, depersonalization, reduced personal accomplishment), the stress system (task stress, role stress, interpersonal stress), the social support and resources system (significant-other support, family support, friend support, self-efficacy, physical activity), and the conflict and sleep system (work interference with family, family interference with work, sleepiness). This assignment is grounded in established frameworks, including Maslach’s burnout model, multidimensional job stress, and conservation of resources theory. To empirically validate this theoretical structure, we conducted a data-driven community detection analysis using the Louvain algorithm (implemented via the igraph package). The algorithm identified four communities with substantial convergence to our theoretical groupings (modularity = 0.527), particularly verifying the distinct clustering of the burnout and stress systems, thereby supporting the construct validity of the theoretical partitioning used in bridge centrality calculations. To further examine sensitivity to community assignment, bridge centrality was also recalculated under an alternative five-community structure separating self-efficacy and physical activity into a distinct “behavioral resources” community; the primary results remained robust. For centrality analysis, four standardized centrality indices—strength, betweenness, closeness, and expected influence—were computed, and bridge strength and bridge expected influence, as proposed by Jones et al. (22), were calculated to identify key bridge nodes connecting different communities. Network robustness was examined using two bootstrap procedures (1,000 resamples each) (19): the non-parametric bootstrap was used to estimate confidence intervals for edge weights, and the case-dropping bootstrap was used to compute the correlation stability coefficient (CS-coefficient), with a CS-coefficient of at least 0.25 considered acceptable and 0.50 or above considered ideal. It is important to note that the edges in the estimated network represent partial correlations, meaning the association between two variables after statistically controlling for all other variables in the network. These partial correlations do not imply causal relationships or temporal precedence. The network should be interpreted as a descriptive model of the conditional dependence structure among the variables, not as a causal or directional model. Causal inference requires longitudinal or experimental designs. All analyses were conducted in R version 4.4.1, primarily using the *bootnet*, *qgraph*, and *networktools* packages.

## 3 Results

### 3.1 Descriptive statistics

Table 1 presents the descriptive statistics for the 14 psychological and behavioral variables among the 637 firefighters.

**Table 1:** Descriptive statistics for all variables (*N* = 637).

Variable	Abbreviation	*M*	*SD*	Min	Max
Task and control stress	TaskStr	12.69	5.71	5	25
Role and development stress	RoleStr	7.85	3.47	3	15
Interpersonal and emotional depletion stress	InterStr	10.24	4.72	4	20
Emotional exhaustion	Exhaus	25.53	14.33	9	63
Depersonalization	Depers	12.21	7.98	5	35
Reduced personal accomplishment	Accomp	31.57	11.15	8	51
Sleepiness	Sleep	9.53	6.47	0	24
Self-efficacy	SelfEff	3.29	0.68	1	4
Work interference with family	WIF	24.64	10.19	9	45
Family interference with work	FIW	22.97	10.27	9	45
Significant-other support	OtherSup	14.11	4.05	4	20
Family support	FamSup	14.80	4.08	4	20
Friend support	FriSup	14.62	4.03	4	20
Physical activity	PhyAct	12.16	11.20	1	48

### 3.2 Network structure

**Figure 1:**
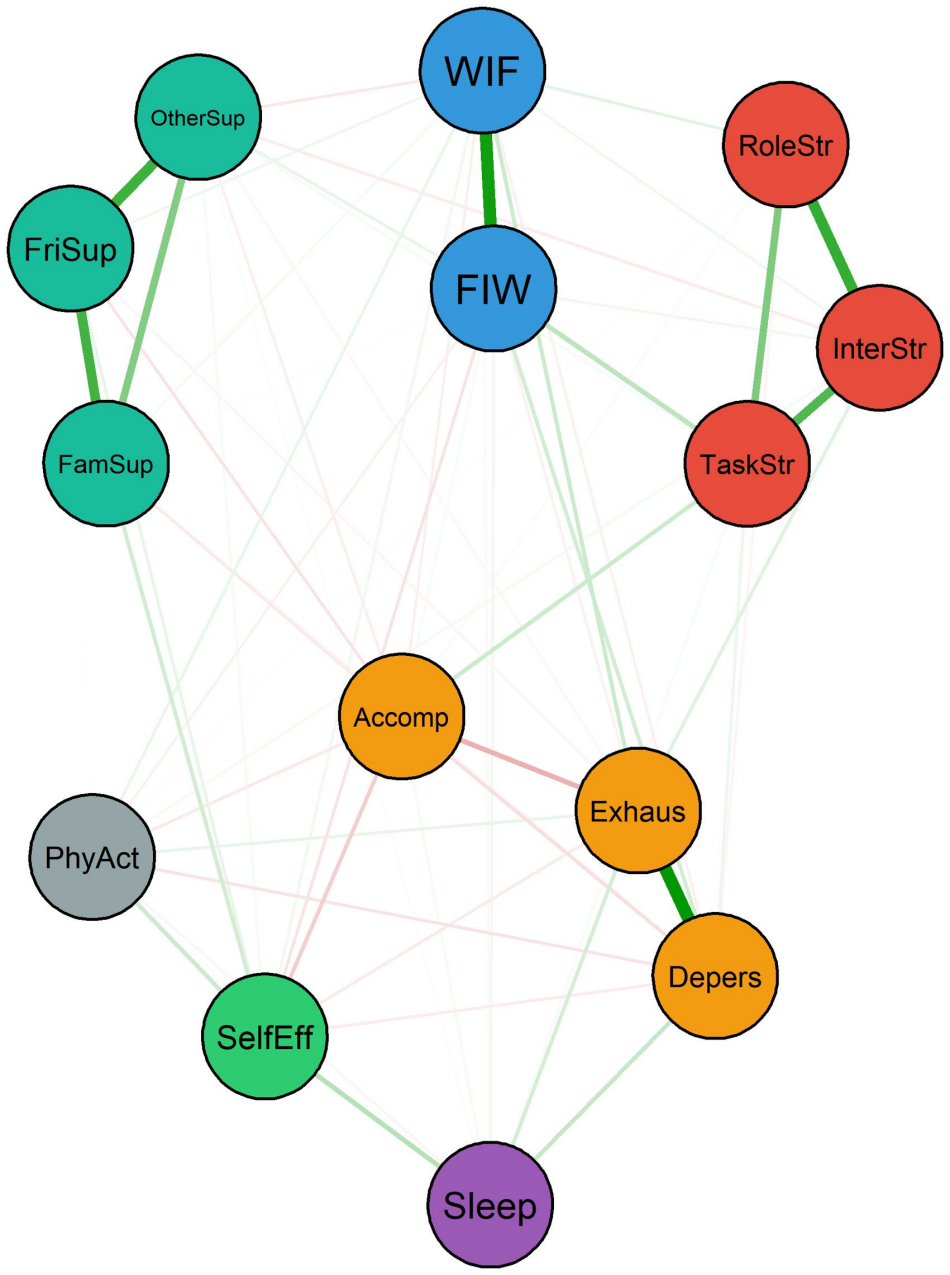
Network structure of psychological and behavioral variables in firefighters.

Figure 1 presents the EBICglasso regularized partial correlation network of the 14 psychological and behavioral variables among firefighters. Of the 91 possible edges in the network, 27 weak connections were shrunk to zero following LASSO regularization (optimal tuning parameter *λ* = 0.0095), retaining 64 edges of substantive significance. The retained edge weights ranged from −0.204 to 0.676.

Table 2 presents the top 10 edges with the largest edge weights and their partial correlation coefficients.

**Table 2:** Top 10 edge weights in the network.

Rank	Node 1	Node 2	Edge weight
1	Emotional exhaustion	Depersonalization	0.676
2	Work interference with family	Family interference with work	0.633
3	Interpersonal stress	Role stress	0.535
4	Significant-other support	Friend support	0.512
5	Family support	Friend support	0.499
6	Task stress	Interpersonal stress	0.461
7	Task stress	Role stress	0.341
8	Significant-other support	Family support	0.329
9	Emotional exhaustion	Reduced personal accomplishment	−0.204
10	Sleepiness	Self-efficacy	0.190

The network structure exhibited clear community clustering characteristics. Within the burnout system, the connection between emotional exhaustion and depersonalization was the strongest (weight = 0.676), forming the core dyad of this system. Reduced personal accomplishment was negatively connected to both (weight with emotional exhaustion = −0.204; weight with depersonalization = −0.083). Within the job stress system, the three dimensions formed a tightly connected positive triangle, with the connection between interpersonal stress and role stress being the strongest (weight = 0.535). Within the social support system, significant-other support, family support, and friend support constituted a strongly connected support subnetwork, with all pairwise edge weights exceeding 0.329. The two directions of work–family conflict (WIF and FIW) were linked by a very strong positive connection (weight = 0.633).

Notable cross-system connections included: emotional exhaustion and work interference with family (weight = 0.135), emotional exhaustion and sleepiness (weight = 0.111), family interference with work and task stress (weight = 0.171), self-efficacy and physical activity (weight = 0.133), and self-efficacy and family support (weight = 0.120). It is worth noting that depersonalization and family interference with work showed a positive connection (weight = 0.118), while self-efficacy and sleepiness exhibited a positive connection (weight = 0.190), suggesting that individuals with lower self-efficacy may also experience higher levels of sleepiness.

### 3.3 Centrality analysis

**Figure 2:**
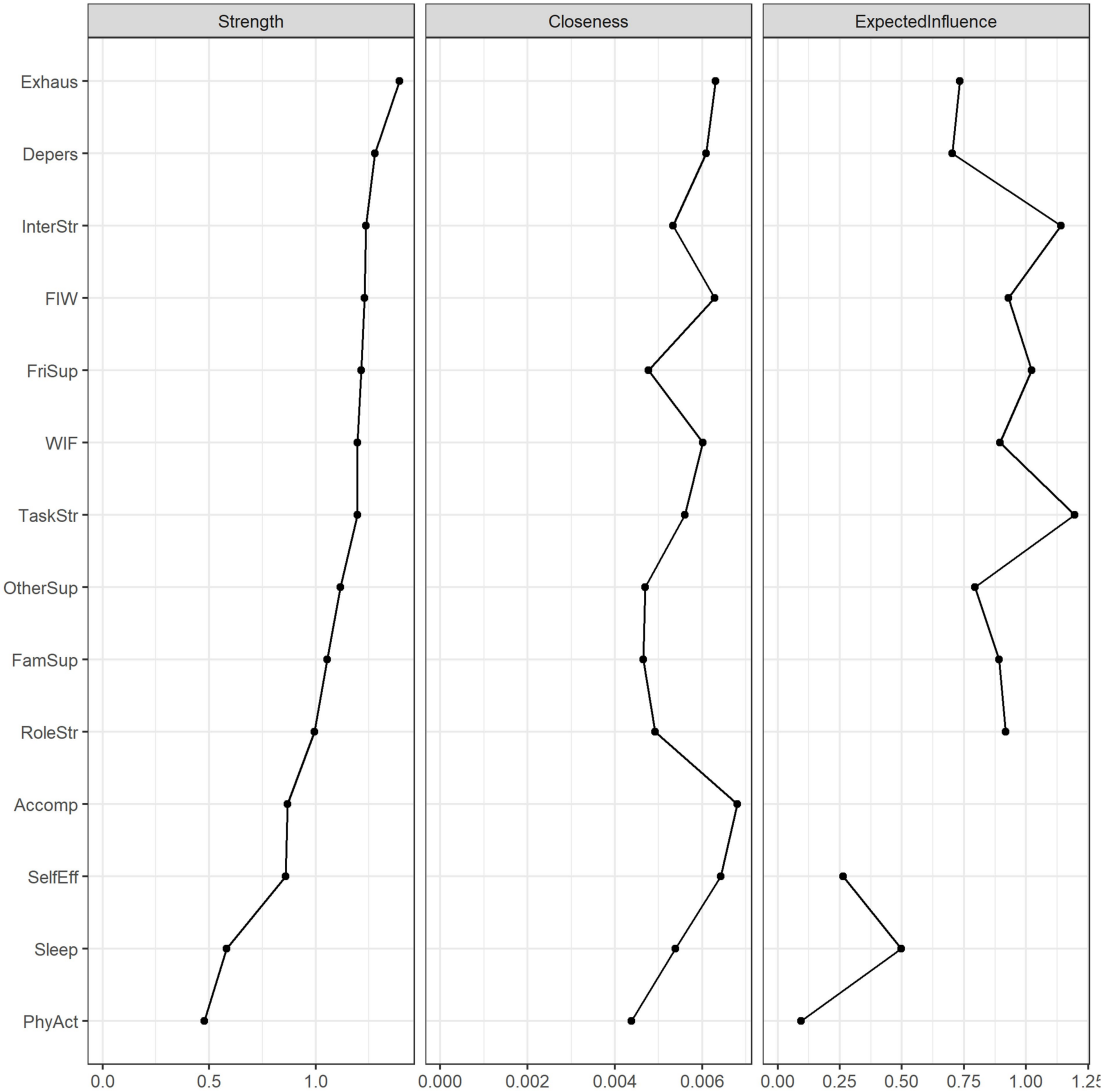
Centrality indices for all nodes (excluding betweenness centrality).

Figure 2 displays the rankings of the 14 nodes across three standardized centrality indices (betweenness centrality is excluded due to low stability).

Strength centrality most reliably identified emotional exhaustion as the network’s core node. Strength centrality analysis indicated that emotional exhaustion (1.29) had the highest overall connectivity strength in the network, indicating that it was most extensively and tightly associated with other variables. Depersonalization (0.85) and interpersonal stress (0.70) ranked second and third, respectively. This implies that changes in emotional exhaustion is most robustly associated with other nodes in the network, making it a key central indicator within the psychological problem network. Physical activity (−2.14) and sleepiness (−1.75) had the lowest strength centrality, indicating that these two variables had relatively few direct connections in the network.

Task stress and interpersonal stress demonstrated the highest potential to co-occur with other network variables. Regarding expected influence, task stress (1.11) exhibited the highest expected influence, followed by interpersonal stress (0.99) and friend support (0.73), indicating that these variables demonstrated the highest net positive Expected Influence, that is, higher levels of these variables tend to strongly co-occur with higher levels of other variables in the network. Reduced personal accomplishment (−2.58) had the lowest expected influence (the negative value with the largest absolute magnitude), reflecting its predominantly negative connections with other nodes. Bootstrapped pairwise difference tests for strength centrality are provided in Supplementary Figure S1.

The results also showed that the correlation stability (CS) coefficient for betweenness centrality was 0.13, falling below the minimum acceptable threshold of 0.25 recommended for network analysis. Therefore, betweenness centrality values are presented in Table 3 for completeness but should not be substantively interpreted, as the metric is highly sensitive to sampling fluctuations in the current network.

**Table 3:** Standardized centrality indices for all nodes.

Node	Strength	Betweenness	Closeness	Expected influence
Emotional exhaustion	1.29	0.80	0.97	0.11
Depersonalization	0.85	−0.13	0.70	0.04
Interpersonal stress	0.70	−0.32	−0.28	0.99
Family interference with work	0.67	−0.32	0.94	0.53
Friend support	0.61	−0.13	−0.99	0.73
Work interference with family	0.55	−0.89	0.60	0.45
Task stress	0.55	0.99	0.07	1.11
Significant-other support	0.25	0.05	−1.10	0.24
Family support	0.02	−0.13	−1.15	0.45
Reduced personal accomplishment	−0.68	2.12	1.61	−2.58
Role stress	−0.21	−1.26	−0.80	0.51
Self-efficacy	−0.71	1.37	1.12	−0.90
Sleepiness	−1.75	−0.89	−0.20	−0.40
Physical activity	−2.14	−1.26	−1.49	−1.27

Betweenness centrality CS-coefficient = 0.13, below the acceptable threshold; interpret with caution.

### 3.4 Bridge centrality analysis

**Figure 3:**
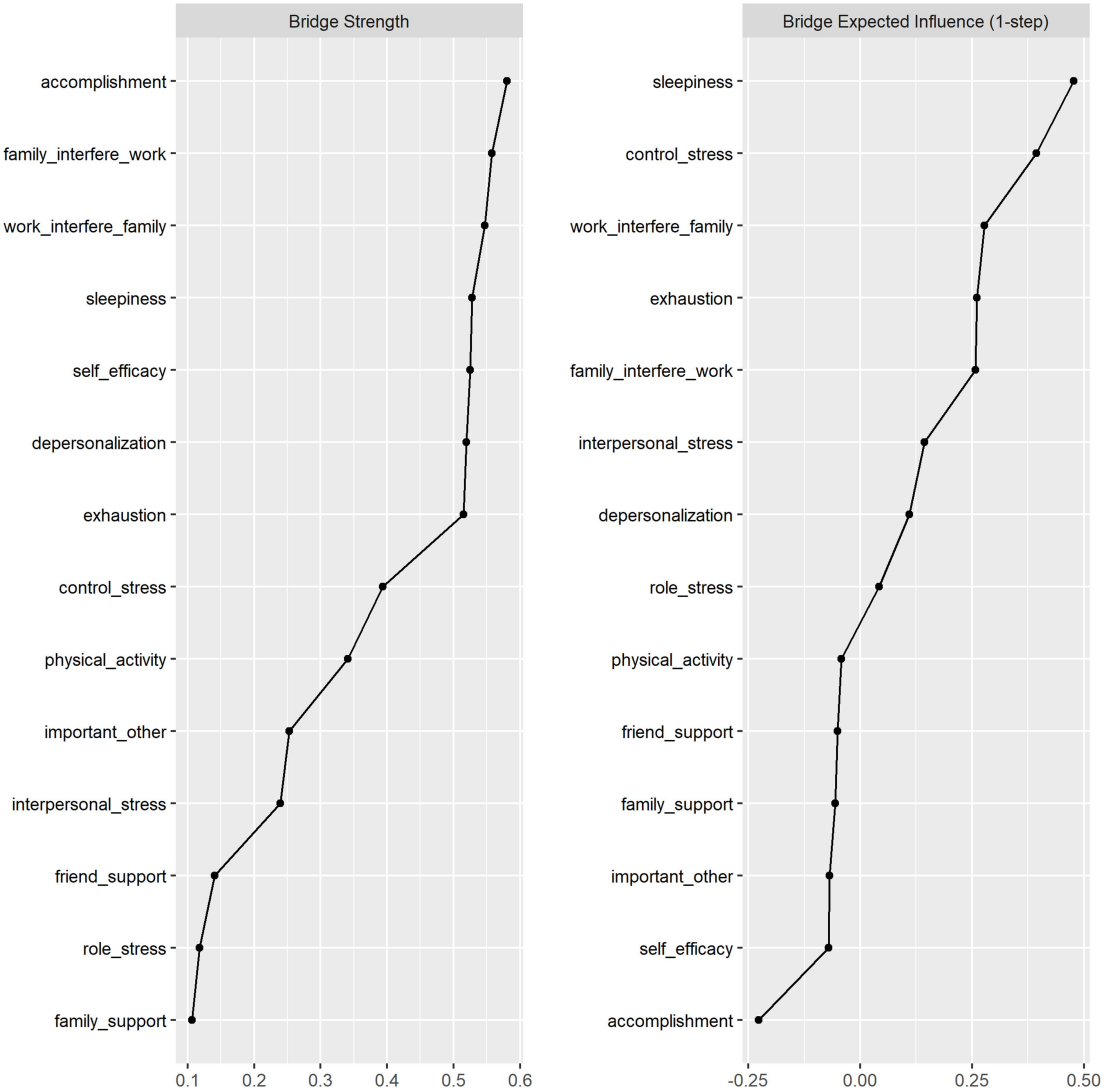
Bridge centrality indices for all nodes.

Figure 3 displays the bridge centrality indices for all nodes.

A cluster of variables, notably reduced personal accomplishment, work–family conflict, sleepiness, and self-efficacy, served as the primary cross-system connectors. Bridge strength analysis (Table 4) indicated that reduced personal accomplishment (bridge strength = 0.581), family interference with work (0.558), work interference with family (0.547), sleepiness (0.528), and self-efficacy (0.525) were the top bridge variables, exhibiting strong overall connectivity with variables outside their respective communities.

**Table 4:** Bridge centrality indices for all nodes.

Node	Bridge strength	Bridge expected influence
Reduced personal accomplishment	0.581	−0.227
Family interference with work	0.558	0.258
Work interference with family	0.547	0.278
Sleepiness	0.528	0.477
Self-efficacy	0.525	−0.07
Depersonalization	0.519	0.11
Emotional exhaustion	0.515	0.261
Task stress	0.393	0.393
Physical activity	0.341	−0.042
Significant-other support	0.253	−0.069
Interpersonal stress	0.239	0.144
Friend support	0.141	−0.051
Role stress	0.118	0.042
Family support	0.106	−0.056

Elevated sleepiness levels exhibited the strongest net positive cross-system associations. Bridge expected influence analysis further revealed the directionality of cross-system influence. Sleepiness (bridge EI = 0.477) ranked first, indicating that elevated sleepiness levels exerted the greatest net positive associations with other systems (particularly the burnout and stress systems). Task stress (0.393) and work interference with family (0.278) also exhibited high bridge expected influence. Notably, the bridge expected influence of reduced personal accomplishment was negative (−0.227), suggesting its cross-system influence was predominantly inhibitory.

Integrating both bridge strength and bridge expected influence indices, self-efficacy and sleepiness emerged as the two most critical bridge variables in the present network, each connecting the burnout, stress, social support, and work–family conflict systems through different mechanisms.

### 3.5 Network robustness

Non-parametric bootstrap results (1,000 resamples) showed that the 95% confidence intervals for the major edges in the network were relatively narrow, indicating good precision in edge weight estimation. The confidence intervals for edges with larger weights (e.g., emotional exhaustion–depersonalization, WIF–FIW, interpersonal stress-role stress) were narrow and did not overlap with zero, confirming the robustness of these connections. Edge weight difference tests supported significant differences among the major edge weights, indicating that the ranking of edge weights in the network was reliable. A plot of bootstrap confidence intervals for all retained edges is provided as Supplementary Figure S2.

Case-dropping bootstrap results (1,000 resamples) yielded CS-coefficients for the four centrality indices as presented in Table 5. The CS-coefficients for strength centrality and expected influence both reached 0.75, far exceeding the ideal benchmark of 0.50, indicating that the ranking of these two indices remained stable even when up to 75% of the sample was randomly dropped, allowing for reliable interpretation in subsequent discussions. The CS-coefficient for closeness centrality was 0.44, exceeding the minimum acceptable threshold of 0.25, though results should be interpreted with caution. The CS-coefficient for betweenness centrality was 0.13, falling below the minimum interpretable threshold of 0.25; this is attributable to the inherent sensitivity of this index to sampling fluctuations, a phenomenon commonly observed in psychological network analyses.

**Table 5:** CS-coefficients for centrality indices.

Centrality index	CS-coefficient	Interpretation
Strength centrality	0.75	Excellent
Expected influence	0.75	Excellent
Closeness centrality	0.44	Acceptable
Betweenness centrality	0.13	Below recommended threshold

## 4 Discussion

Using a sample of 637 Chinese firefighters and incorporating 14 psychological and behavioral variables spanning occupational burnout, job stress, work–family conflict, social support, sleepiness, self-efficacy, and physical activity, this study constructed an EBICglasso regularized partial correlation network. To orient the reader before contextualizing these findings within the broader literature, the most clinically meaningful results are highlighted as follows: (1) the strongest edge in the network was between emotional exhaustion and depersonalization, indicating these burnout components are tightly coupled. (2) Emotional exhaustion was the most central node by strength centrality, suggesting it possesses the broadest associative reach in the psychological problem network. (3) Sleepiness and self-efficacy emerged as critical cross-system connectors, with sleepiness exhibiting the highest bridge expected influence. These core findings provide the empirical backbone for the subsequent interpretation and the formulation of targeted, hypothesis-generating intervention strategies.

### 4.1 Network structure characteristics

The network analysis results demonstrated that firefighters’ psychological and behavioral variables formed a network topology with distinct community clustering features. The burnout, stress, social support, and conflict–sleep systems each constituted relatively tightly connected subnetworks while being interconnected through specific cross-system edges. This finding is consistent with the network theory of mental disorders proposed by Borsboom (18), which posits that psychological problems interact directly with one another rather than merely sharing a common latent cause. The highest edge weight was observed between emotional exhaustion and depersonalization (0.676), a finding consistent with a substantial body of prior research. In Maslach’s stage model of burnout development, emotional exhaustion is considered the initial response in the burnout process: individuals first experience depletion of emotional resources under sustained work pressure and subsequently adopt detached and cynical attitudes toward work recipients (depersonalization) to protect their remaining psychological resources, resulting in a tight cascading relationship between the two (33). Liu et al. (14) similarly identified emotional exhaustion as the core component of the burnout network in a Chinese firefighter sample, further supporting the primacy and driving role of emotional exhaustion in the burnout development process. The strong connection between work interference with family and family interference with work (0.633) reflects the bidirectional cyclical nature of work–family conflict. The role conflict theory proposed by Greenhaus and Beutell (9) holds that work and family are two role domains that compete for time, energy, and attention, and the higher the role demands of one domain, the more it interferes with role fulfillment in the other. Among firefighters, frequent shift rotations and emergency deployments lead to work interfering with family life, while unmet responsibilities and conflicts at home may spill back into the work domain, forming a vicious cycle. This tight bidirectional connection suggests that interventions addressing firefighters’ work–family conflict need to attend to both directions simultaneously. The tight connections among the three job stress dimensions (interpersonal stress–role stress: 0.535; task stress–interpersonal stress: 0.461) indicate that firefighters’ stress experience is highly systemic—stressors from different sources tend to co-occur and mutually reinforce one another. This is consistent with the findings of Harvey et al. (1), who noted that the job demands of firefighters are multidimensional and that improvement in a single dimension of stress may be insufficient to produce a substantive effect. The strong connections among the three sources within the social support system (significant other–friend: 0.512; family–friend: 0.499; significant other–family: 0.329) suggest that different sources of social support are highly synergistic, which may reflect that individuals with well-developed social networks tend to report higher levels across all support sources. Osman et al. (34) noted high correlations among the three first-order factors in their study of the measurement invariance of the MSPSS.

### 4.2 Central nodes

The centrality analysis in this study consistently identified emotional exhaustion as the most central node in the network. Emotional exhaustion ranked first in strength centrality (1.29), indicating that it was most extensively and tightly associated with other variables in the network. This finding is highly consistent with prior literature regarding the central role of emotional exhaustion in the burnout process. Demerouti et al. (27) explicitly stated in the JD-R model that job demands are primarily associated with the exhaustion dimension and that exhaustion is the core component of burnout. Leiter and Maslach (33) also emphasized that among the three dimensions of burnout, emotional exhaustion is the most prominent clinical feature and serves as one of the key indicators for identifying burnout states in latent profile analysis. The reason emotional exhaustion occupies such a central position in the multivariable network may be related to its role as a confluence point for multiple psychological and behavioral problems. On one hand, emotional exhaustion directly reflects the sustained depletion of psychological resources during the process of coping with job stress; on the other hand, it maintains direct connections with multiple cross-system variables such as sleep problems (weight = 0.111), work–family conflict (weight = 0.135), and personal accomplishment (weight = −0.204). Dyal et al. (6) identified a complex bidirectional relationship between sleep health and occupational burnout among firefighters, and emotional exhaustion may serve as a key linking node between the two. Recent evidence from Korean workers demonstrated that burnout—particularly the exhaustion sub-dimension—mediated approximately 51% of the effect of occupational stress on health-related productivity loss (35). Its demonstration that exhaustion serves as a primary functional conduit from stress to downstream outcomes provides convergent support that aligns with the network centrality findings reported here. The specific occupational demands of firefighting amplify the mechanisms underlying emotional exhaustion’s centrality. Unlike many occupational groups, firefighters are simultaneously exposed to acute traumatic events (e.g., fatality scenes, severe injuries), chronic low-grade stress (e.g., unpredictable deployment schedules, prolonged standby periods), and role ambiguity arising from evolving rescue mandates. This layered stress environment means that emotional resources are depleted not only during active operations but throughout the standby period, making the emotional exhaustion–depersonalization dyad structurally dominant in a way that may be specific to this occupational group. The tight coupling between emotional exhaustion and work–family conflict (weight = 0.135) further reflects a firefighter-specific mechanism: 24-h shift rotations disrupt both sleep rhythms and family routines simultaneously, creating a reinforcing cycle of exhaustion and role conflict that generic stress management programs, which was designed for standard working-hour populations, may fail to adequately address. Depersonalization, as the node with the second-highest strength centrality (0.85), also merits attention. Depersonalization in the firefighter population manifests as emotional detachment from those seeking help and from colleagues, which not only compromises team collaboration and rescue effectiveness but may further exacerbate individuals’ sense of isolation and occupational dissatisfaction (2). Interpersonal stress ranked third (0.70), suggesting that interpersonal aspects of job stress represent another important activation point in the firefighters’ psychological problem network, consistent with the highly team-dependent nature of firefighting work. It is critical to acknowledge that the central position of emotional exhaustion in this network does not establish it as a cause of other psychological problems. Cross-sectional network analysis cannot determine temporal precedence: emotional exhaustion may precede and drive other symptoms, or it may itself be an outcome of accumulated stress, sleep deprivation, and work–family conflict. For example, while Maslach’s stage model of burnout development theorizes that exhaustion precedes depersonalization, network edges here only reflect that the two variables are strongly associated after controlling for all others (36). Future longitudinal or experience-sampling studies are needed to establish whether emotional exhaustion functions as a predictor, consequence, or both within this variable system.

Additionally, the moderate bridge centrality of social support nodes warrants a nuanced interpretation. While social support is widely theorized as a stress buffer, recent evidence suggests its protective effects are not universal. For instance, Zhou et al. (37) found that social support could paradoxically intensify stress pathways under certain conditions, revealing that resilience factors do not uniformly buffer the impact of adversity and may amplify stress depending on contextual moderators. In our firefighter network, social support operates primarily within its own subsystem and does not consistently bridge toward burnout, which aligns with the premise that support-based resources carry contextual boundary conditions.

### 4.3 Bridge variables

Bridge centrality analysis constitutes a novel contribution of the present study. Rather than a single dominant bridge node, the results revealed a cluster of highly connected bridge variables including reduced personal accomplishment, family/work interference, sleepiness, and self-efficacy. These variables collectively connect the burnout, stress, social support, and behavioral systems. Although not the single highest bridge node, self-efficacy remains a critical hub. Our finding that general self-efficacy serves as a critical cross-system bridge aligns with recent network literature highlighting the bridging role of positive cognitive resources. For instance, Li et al. (2025) demonstrated that positive future-oriented beliefs (e.g., hopefulness, a construct conceptually closely tied to self-efficacy) function as key bridge nodes linking distinct psychological systems, such as parental involvement and depressive symptoms (38). This underscores the role of generalized cognitive resources like self-efficacy not merely as isolated traits, but as robust buffers that span across specific symptom clusters. The role of self-efficacy as a bridge variable can be understood from multiple theoretical perspectives. First, according to Bandura’s (15) social cognitive theory, self-efficacy regulates the stress-coping process through its influence on individuals’ cognitive appraisal, affective responses, and behavioral choices. Firefighters with high self-efficacy are more likely to appraise high-pressure environments as challenges rather than threats and are more likely to adopt proactive coping strategies (such as engaging in physical exercise), thereby more effectively alleviating stress and emotional exhaustion (16). Second, the connections between self-efficacy and the social support system (with family support: 0.120; with friend support: 0.059) suggest that the maintenance and strengthening of self-efficacy may partly depend on positive social interactions. Support and encouragement from family and friends not only provide an emotional buffer but can also directly enhance individuals’ confidence in coping with difficulties, creating a virtuous cycle of mutual reinforcement in resource acquisition (39). Indeed, recent longitudinal evidence confirms that self-efficacy and future orientation are prospectively associated with distinct sources of support (40). Furthermore, examining social support at a source-specific level (e.g., family vs. friends), as the present study does, is theoretically crucial, because distinctive sources of support carry different implications for psychosocial well-being (41). Moreover, recent person-centered research has highlighted that the protective function of social support depends heavily on individual dispositional configurations (42). This nuance underscores why assigning self-efficacy and social support to the same community warrants critical examination, and why our sensitivity analysis (which retained the robustness of self-efficacy as a top bridge node even when separated from social support) is theoretically informative. Third, the direct connection between self-efficacy and physical activity (0.133) indicates that it serves as a critical hub node linking psychological states (such as stress and burnout) with behavioral outcomes (such as actual exercise and proactive coping).

Importantly, sleepiness ranked first in bridge expected influence (0.477), indicating that elevated sleepiness levels exhibit the strongest net positive associations with other systems (particularly the burnout and stress systems). This finding is consistent with the research of Barger et al. (13) and Dyal et al. (6), who found tight bidirectional associations between sleep problems and emotional exhaustion and job stress among firefighters. The role of sleepiness as a bridge variable can be understood from the perspective of the conservation of resources theory (43): sleep insufficiency directly accelerates the depletion of physiological and psychological resources, making individuals more susceptible to emotional exhaustion (the subjective experience of resource loss), while simultaneously reducing their capacity to perceive and utilize social support and their efficacy in coping with job stress (44, 45). Among firefighters, sleepiness is not merely a generic health concern but a structurally embedded occupational hazard. Irregular 24-h shifts, nighttime emergency deployments, and high-alert standby states fragment sleep architecture in ways qualitatively different from ordinary shift work (13). This firefighter-specific sleep disruption pattern may explain why sleepiness functions as a cross-system bridge rather than an isolated symptom: chronic sleep fragmentation impairs emotional regulation capacity (increasing vulnerability to emotional exhaustion), reduces perceived social support effectiveness (as fatigued individuals withdraw from social interactions), and diminishes exercise motivation (undermining self-efficacy). Interventions designed specifically for the firefighting context, such as strategic napping policies during night shifts, light-exposure management in station environments, and CBT-I protocols adapted for shift workers, may therefore have disproportionately broad effects on the overall psychological problem network. Notably, although sleepiness had relatively low strength centrality (−1.75), its bridge centrality was high—this pattern of “low overall connectivity but high cross-system connectivity” precisely embodies the distinctive nature of bridge variables: they are not necessarily the most densely connected nodes in the network but serve as critical channels crossing system boundaries (22). This also implies that relying solely on traditional centrality indices may overlook the important role of sleepiness in the cross-system transmission of psychological problems, highlighting the unique value of bridge centrality analysis in comprehensive psychological network research. Finally, the positive partial correlation between sleepiness and self-efficacy (weight = 0.190) warrants careful interpretation. Three mechanisms may account for this counterintuitive direction. First, suppressor effects may be operating: in partial correlation networks, edge directions can differ from bivariate correlations because shared variance with other network variables is controlled. Second, this may reflect an occupational phenomenon specific to high-stress rescue workers: individuals with high general self-efficacy (“I can persist through setbacks”) often deploy effortful over-engagement to cope with extreme job demands, maintaining high operational engagement that ultimately contributes to elevated daytime sleepiness. Third, Bertrand et al. (2025) demonstrated in a nine-country longitudinal study that physical activity predicted well-being through broad enhancement rather than fatigue-specific pathways, suggesting the relationship between efficacy-driven behaviors and fatigue is complex (46). Future studies should include overtraining or workaholism metrics to disentangle these mechanisms.

### 4.4 Potential targets for future interventions

An important caveat applies to the intervention implications discussed below: centrality and bridge indices reflect the statistical structure of partial correlations in this cross-sectional sample, not confirmed causal relationships. Consequently, highly central or bridging nodes should not be treated as confirmed intervention targets but rather as hypothesis-generating candidates that warrant prioritization in future longitudinal and experimental research. With this caveat in mind, the present network analysis offers three directions that may inform the design of future intervention studies.

First, emotional exhaustion emerges as a highly prioritized candidate for future intervention studies. Given its highest strength centrality, it represents a core feature of the network, suggesting that its mitigation may be closely tied to the overall improvement of the network state. In practice, future research could explore whether firefighters’ emotional exhaustion levels may be reduced through mindfulness-based stress reduction (MBSR), emotion regulation skills training, and optimization of shift scheduling systems. Second, enhancing self-efficacy could serve as a potential pathway to mitigate the co-occurrence of cross-system psychological problems. As a key bridge variable linking multiple domains, investigating self-efficacy interventions may provide insights into simultaneous improvements across the burnout and stress systems. Third, improving sleep quality constitutes another intervention entry point with leveraged effects. As the variable with the highest bridge expected influence, improvement in sleepiness could be a valuable focus for addressing its strong positive associations with the burnout and stress systems. Applicable measures for future testing include: optimizing shift scheduling to minimize disruption to circadian rhythms, conducting sleep hygiene education, introducing cognitive behavioral therapy for insomnia (CBT-I), and providing sleep-conducive environmental conditions at fire stations. Overall, focusing on emotional exhaustion could be explored as a way to address a central feature of the network, while investigating the roles of self-efficacy and sleep improvement in longitudinal studies may provide insights into mitigating cross-system associations, potentially contributing to systemic improvements in firefighter mental health.

### 4.5 Limitations and future directions

This study has the following limitations. First, the cross-sectional design limits causal inference. The edge weights in the network reflect partial correlations rather than causal relationships, and the causal directions among variables require further verification through longitudinal or experimental studies. Future research could employ the experience sampling method to collect intensive longitudinal data and construct within-person temporal networks to reveal temporal precedence and causal directions among variables. Furthermore, as recently demonstrated by McNeill and Cullington (47), relationships between workload, work-life conflict, and stress are highly dependent on individual moderating factors. Such contextual dependencies cannot be fully captured in cross-sectional network topology, meaning our intervention implications should be treated as hypotheses awaiting longitudinal validation rather than prescriptive guidelines. Second, all measurement instruments were self-report scales, posing a risk of common method bias. Future research could incorporate objective indicators (e.g., sleep and physical activity data collected via wearable devices, physiological stress biomarkers such as cortisol levels) to provide cross-validation through multi-source data. Third, the sample was drawn from Chinese firefighters, and the high concentration of urban-registered personnel may limit the generalizability of the findings to firefighters in specialized rural or remote stations. Future research should replicate this study across different countries, cultural contexts, and regional station types to examine the universality and specificity of the network structure. Fourth, longitudinal data tracking mental and somatic ill health as long-term predictors of burnout symptoms (48) would be required to establish whether social support nodes and bridge variables play true causal buffering roles.

## 5 Conclusion

This study employed network analysis to systematically reveal the complex associative structure among 14 psychological and behavioral variables in firefighters. The main conclusions are as follows: (1) firefighters’ psychological and behavioral variables formed a network structure with distinct community clustering, in which the burnout, stress, social support, and conflict–sleep systems each constituted tightly connected subnetworks. (2) Emotional exhaustion was the most central node in the network, most tightly connected with depersonalization, and represented the most prominent core feature of the psychological problem network. (3) Sleepiness and self-efficacy were among the most important bridge variables connecting different psychological systems, playing critical channel roles in linking distinct systems of psychological problems. (4) Future intervention studies and longitudinal research may consider targeting emotional exhaustion, self-efficacy, and sleep quality as prioritized candidates to potentially mitigate cross-system comorbidities among firefighters. This study provides network analysis-based empirical evidence for the precise assessment and stratified intervention of firefighter mental health.

## Data Availability

The raw data supporting the conclusions of this article will be made available by the authors, without undue reservation.

## Supplementary material

SUPPLEMENTARY FIGURE S1 Bootstrapped pairwise difference tests for strength centrality.

SUPPLEMENTARY FIGURE S2 The plot of bootstrap confidence intervals for all retained edges.
