# Supplementary figures and images for: Network analysis of psychological and behavioral variables in firefighters: identification of central nodes and bridge variables
Source: Front Public Health. 2026 Jun 3;14:1846200. doi: 10.3389/fpubh.2026.1846200 (PMC13272169; doi:10.3389/fpubh.2026.1846200)

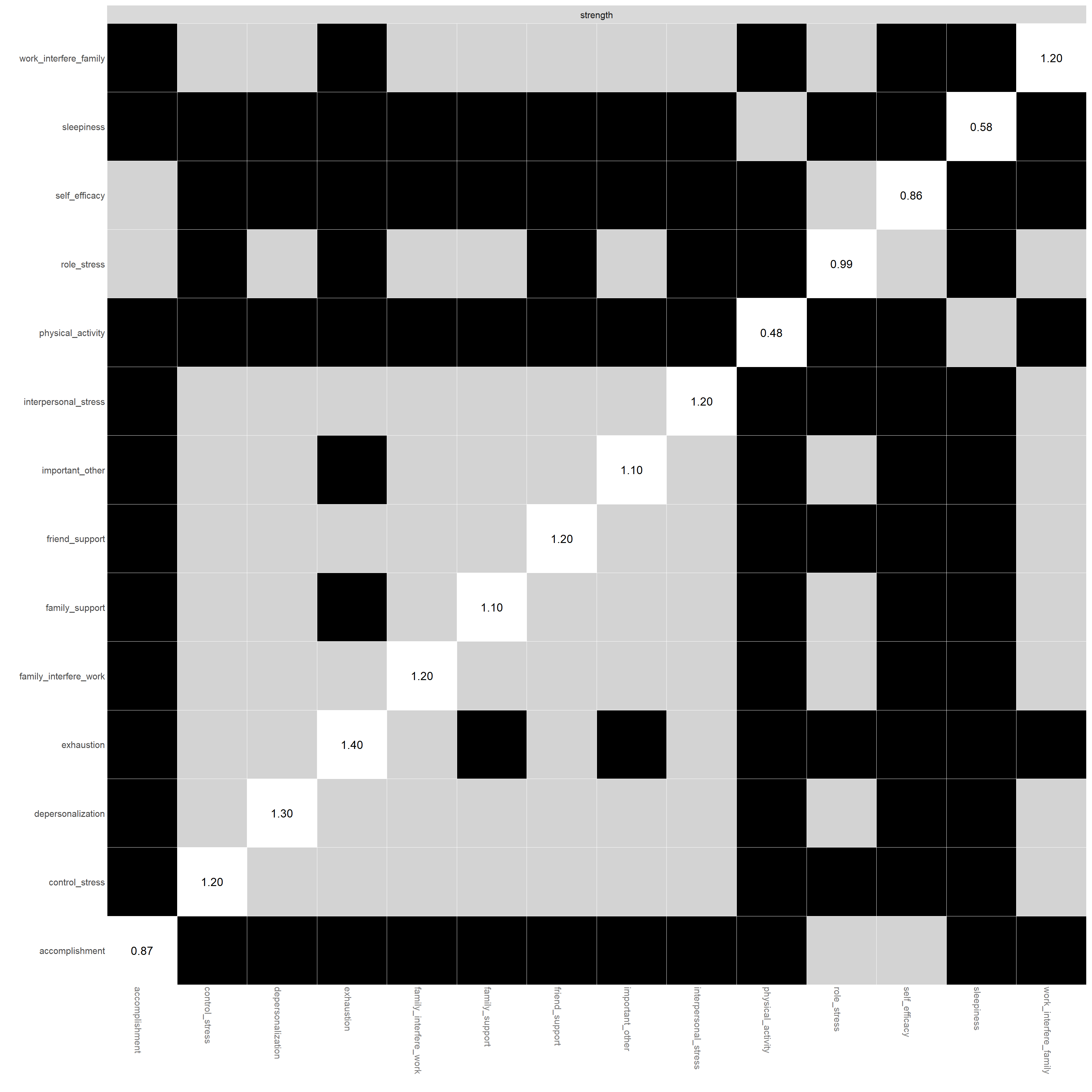

Supplement: SUPPLEMENTARY FIGURE S1 — Bootstrapped pairwise difference tests for strength centrality. [file Image_1.JPEG]

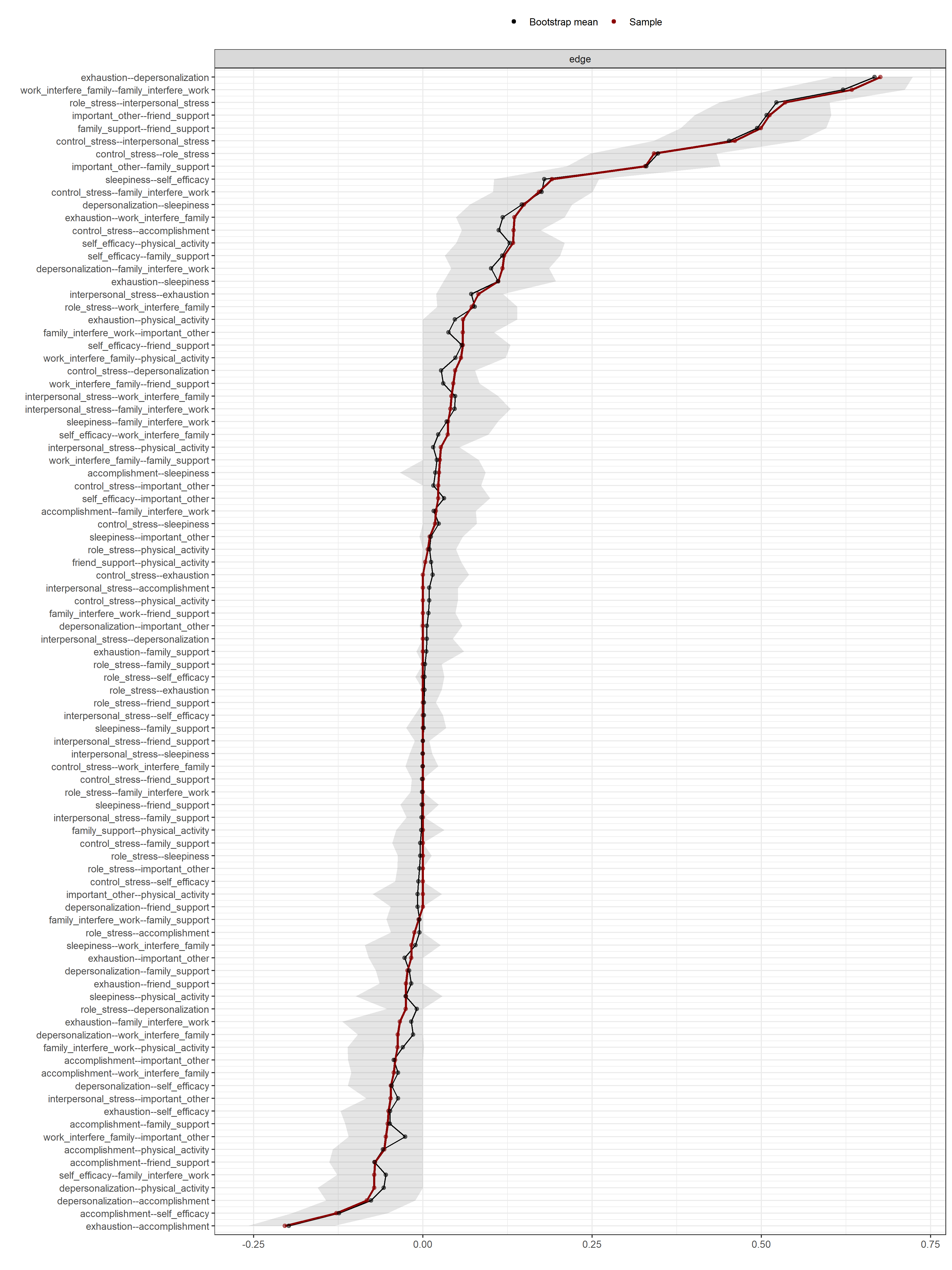

Supplement: SUPPLEMENTARY FIGURE S2 — The plot of bootstrap confidence intervals for all retained edges. [file Image_2.JPEG]
